# RF-EMF Exposure near 5G NR Small Cells

**DOI:** 10.3390/s23063145

**Published:** 2023-03-15

**Authors:** Sam Aerts, Kenneth Deprez, Leen Verloock, Robert G. Olsen, Luc Martens, Phung Tran, Wout Joseph

**Affiliations:** 1 WAVES, Department of Information Technology, Ghent University/imec, 9052 Ghent, Belgium; 2 School of Electrical Engineering & Computer Science, Washington State University, Pullman, WA 99164, USA; 3 Electric Power Research Institute (EPRI), Palo Alto, CA 94304, USA

**Keywords:** 5G new radio, cellular network, exposure assessment, exposure limits, radiofrequency electromagnetic fields, small cell

## Abstract

Of particular interest within fifth generation (5G) cellular networks are the typical levels of radiofrequency (RF) electromagnetic fields (EMFs) emitted by ‘small cells’, low-power base stations, which are installed such that both workers and members of the general public can come in close proximity with them. In this study, RF-EMF measurements were performed near two 5G New Radio (NR) base stations, one with an Advanced Antenna System (AAS) capable of beamforming and the other a traditional microcell. At various positions near the base stations, with distances ranging between 0.5 m and 100 m, both the worst-case and time-averaged field levels under maximized downlink traffic load were assessed. Moreover, from these measurements, estimates were made of the typical exposures for various cases involving users and non-users. Comparison to the maximum permissible exposure limits issued by the International Commission on Non-Ionizing Radiation Protection (ICNIRP) resulted in maximum exposure ratios of 0.15 (occupational, at 0.5 m) and 0.68 (general public, at 1.3 m). The exposure of non-users was potentially much lower, depending on the activity of other users serviced by the base station and its beamforming capabilities: 5 to 30 times lower in the case of an AAS base station compared to barely lower to 30 times lower for a traditional antenna.

## 1. Introduction

An important innovative aspect of the latest generation of cellular networks (i.e., the fifth generation, or 5G) is the widespread deployment of low-powered base stations (referred to as densification), known under the umbrella term ‘small cells’. Small cells are expected to be mounted lower on the infrastructure and with a higher density in order to provide a significant increase in network capacity where it is needed, such as in dense urban environments, sports venues, and shopping malls. As such, both workers (e.g., for utility companies) and members of the general public are more likely to reside in close proximity to these sources of radiofrequency (RF) electromagnetic fields (EMFs).

Regarding the size and technical specifications of small cells, there is no consensus between the various standardization and legislative bodies. For example, for the 3G Partnership Project (3GPP), a base station is a small cell when “the antenna is sited above the median but below the maximum height of the surrounding roof tops” [1]. However, a categorization scheme for base stations based on the equivalent isotropic radiated power (EIRP) was developed by the International Electrotechnical Commission (IEC) [2] and adopted in European Union (EU) legislation [3], found in Table 1. IEC base station classes E100, E10, and E2 fall under the general definition of a small cell.

Small cells may contain a small number of antenna elements to permit up to four spatial layers (i.e., maximum 4 × 4 multiple-input–multiple-output (MIMO)) transmitted sector-wide (i.e., with an opening angle of 120°), or they may comprise Advanced Antenna Systems (AAS), which contain antenna arrays of a large number of antenna elements (up to hundreds) and provide massive MIMO and beamforming capabilities.

However, base stations with an Advanced Antenna System (AAS) are not considered in this E-class categorization scheme. Therefore, in [3], for outdoor AAS with massive multiple-input–multiple-output (MaMIMO) beamforming antennas, an upper limit of 30 dBm (or 1 W) transmit power was suggested instead of the guidelines of Table 1 (which are kept for traditional passive antennas).

In situ measurements of RF-EMF exposure levels near small cells have been performed previously in [4,5] (3G indoors), [6] (2G indoors), [7] (mixed 2G–4G, mixed indoors/outdoors, broadband measurements), and [8] (4G outdoors). The studied small cells featured passive antennas. In addition, maximum power densities at close distances (down to 1 m) of 5G small cells operating at 28 GHz (with AAS) were simulated in [9] for several transmission powers and antenna gains. Furthermore, in situ measurement methodologies for 5G new radio (NR) signals have been presented in, e.g., [2,10,11,12,13] for frequencies below 6 GHz (NR Frequency Range (FR) 1) and in, e.g., [14,15,16] for frequencies above 24 GHz (NR FR2, also called millimeter waves or mmWaves).

This study delivers the first assessment of both worst-case (extrapolated and measured) as well as typical RF-EMF exposure levels near 5G NR small cells, using the measurement methodology developed previously in [10] and adopted by the IEC [2]. The results of this work can be used by, e.g., electric utilities (i.e., companies in the electric power industry) who are considering placing 5G small cells on poles or other utility infrastructures in neighborhoods.

First, the measurement equipment and methods are outlined, followed by a description of the base station sites. Then, typical exposure use cases are introduced, which, along with the extreme use cases with maximum exposures, result in a range of potential exposures induced by 5G NR small cells in the population based on the measurements. Finally, the exposure levels were compared to the reference levels in the guidelines issued by the International Commission on Non-Ionizing Radiation Protection (ICNIRP) [17].

## 2. Materials and Methods

The measurements of the exposure levels in terms near 5G new radio (NR) base stations were performed using two different measurement setups in parallel and following an updated measurement protocol of the one presented in [10].

### 2.1. Measurement Equipment

**Figure 1:**
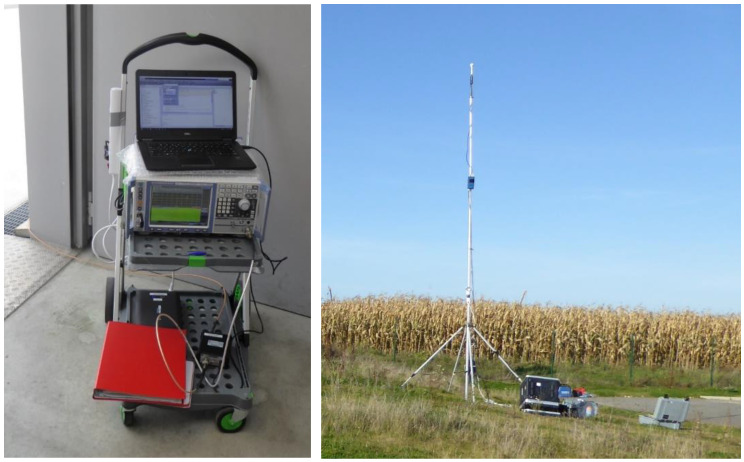
First type of equipment used in this study to measure the exposure to 5G new radio base stations, consisting of an R&S FSV30 spectrum analyzer controlled by a laptop (both on the (**left**)), and an isotropic tri-axial R&S TS-EMF B2 antenna (on the (**right**), placed on a telescopic pole).

The first measurement setup (Figure 1) consisted of a spectrum analyzer (SA) of type Rohde & Schwarz (R&S) (Munich, Germany) FSV30 with a frequency range of 10 Hz to 30 GHz combined with an isotropic tri-axial antenna of type Satimo TSEMF26 (further also called a “probe”) (Microwave Vision Group, Villejust, France). This probe contains three orthogonally arranged antenna elements that are switched electronically, and has a frequency range of 2 GHz to 6 GHz and a dynamic measurement range of 2.5 mV/m to 200 V/m. The FSV30 comes with the ‘spectrogram’ option, which allows a large number of successive measurement samples to be captured. This setup is further called ‘FSV setup’.

**Figure 2:**
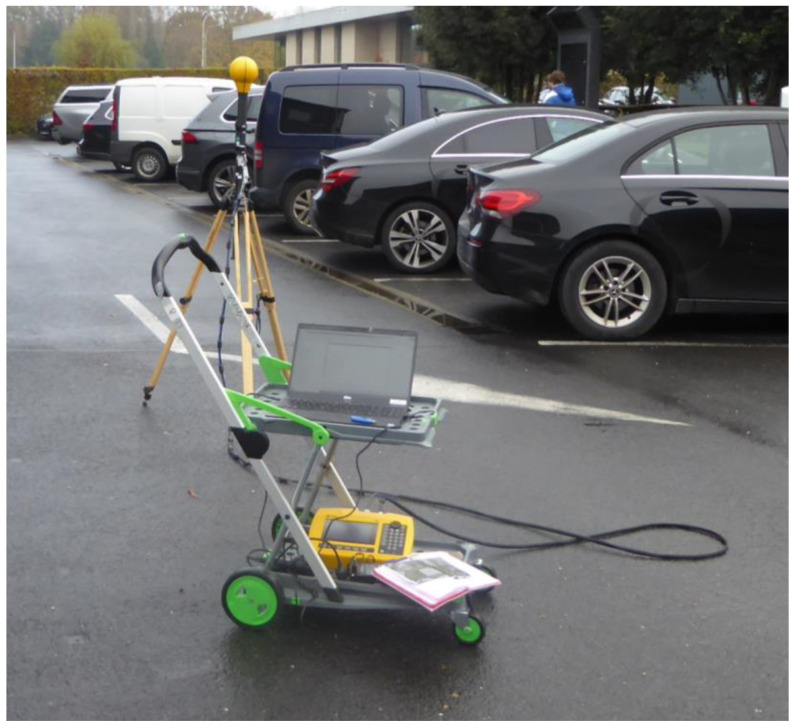
Second type of equipment used in this study to measure the exposure to 5G new radio base stations, consisting of a Narda SRM-3006 field strength analyzer (SRM) and a tri-axial isotropic antenna (“probe”).

The second measurement setup (Figure 2) consisted of a Narda SRM-3006 field strength analyzer (Narda Safety Test Solutions, Pfullingen, Germany) with a frequency range from 9 kHz to 6 GHz combined with an isotropic tri-axial antenna. This probe has a frequency range of 420 MHz to 6 GHz and a dynamic measurement range of 0.14 mV/m to 160 V/m. This setup is further called ‘SRM setup’.

Between the measurement setups, the SRM setup is the most portable and user-friendly and may be more readily available, but its measurement settings are limited. Additionally, a dedicated 5G NR measurement option for code-selective measurements makes this setup even more useful. However, during this research, the option was not yet available.

Whereas the SRM setup was used for measurements at ground level (1.5 m above the ground) (Figure 2), the FSV setup was simultaneously used for measurements at the height of the base station antenna, with the probe placed on a telescopic pole (Figure 1).

### 2.2. Measurement Method

The followed measurement procedure was presented in [10] and adopted in the revised IEC 62232 standard [2]. The different steps are summarized below, while the specific measurement settings can be found in [13]:

**Step 1.** A spectrum overview measurement of the frequency range between 700 MHz and 6 GHz was performed at one position in the environment of the assessed 5G NR base stations (in this study, at about 5 m horizontal distance) using the aforementioned FSV setup. This measurement identified the RF environment at the specific location, and more specifically the frequency band of the considered NR signal.

**Step 2.** An in-band measurement of the considered NR signal was performed at the same position as Step 1, using the same FSV setup (to the authors’ knowledge, this measurement cannot easily be performed with the SRM setup without the aforementioned 5G NR option). The measurement data (i.e., the received-power samples) were post-processed to retain only those signals (i.e., samples above the noise floor) that are roughly four Orthogonal Frequency-Division Multiplexing (OFDM) symbols long, which is the size of one NR Synchronization Signal Block (SSB) in the time domain. For example, in the case of a subcarrier spacing (SCS) of 30 kHz, the SSB is about 4 × 35.7 µs = 142.8 µs long. Plotting just samples of these four-symbol long signals reveals the specific frequencies used by the SSB, and thus its center frequency (SS_REF_) and its bandwidth (which, in the case of an SCS of 30 kHz, is 7 MHz). In the case SS_REF_ is known, this step can be skipped.

**Step 3.** In this study, at several positions in the near vicinity of the base station as well as at distances of up to 100 m, a scope or zero-span measurement was performed with a resolution bandwidth (RBW) of 1 MHz around SS_ref_. The sample time is set approximately equal to the OFDM symbol duration (e.g., ~36 µs for 30 kHz SCS), such that the obtained samples constituted the root-mean-squared (rms) power *P_r_* received during one OFDM symbol and within 1 MHz, which contains about 33 resource elements in the case of a 30 kHz SCS.

With these measurement settings and for an SCS of 30 kHz, it is known that during the transmission of SSB(s) (at frequencies below 6 GHz there can be up to 8 SSBs transmitted consecutively), the measured 1 MHz bandwidth contained 33 REs that were effectively used, so each SSB’s power per resource element *P_RE,SSB_* can be directly derived from the *P_r_* measured during the SSB transmission [10,18].

However, for other signal types, such as the Physical Downlink Shared Channel (PDSCH), i.e., the downlink transmission of data from the base station to a user equipment (UE), the allocation of the REs is not known. At any given time, the number of allocated REs within the measured bandwidth can vary between 0 and 33 (with SCS = 30 kHz). These different allocations result in different Gaussian distributions making up the total received-power sample distribution [10]. Hence, to obtain the correct *P_RE,PDSCH_*, it is essential that the number of resources allocated to PDSCH is maximized during this measurement, forcing at least sometimes 33 active REs within the measured bandwidth. This is performed by maximizing the downlink traffic load, e.g., by using iPerf, a speed test, or by downloading a very large file (of several GBs) on a UE in the near vicinity of the measurement probe [13]. Then, the ‘maximum’ *P_r_* (actually, the peak of the highest-valued Gaussian distributions) measured during PDSCH transmission is used to calculate *P_RE,PDSCH_* [10].

Since the FSV setup’s probe was put on a telescopic pole, measurements with an active UE involved placing the UE on a plastic cart (~0.8 m above ground level) at a 2 m distance from the SRM setup’s probe.

Before this study, this type of measurement had only been performed with the FSV setup [10,11], with which a large amount of measurement traces with minimal blind time (i.e., the time between traces during which nothing is measured) can be saved (thanks to the spectrogram option). However, in this study, the more practical SRM setup was also used in order to determine its validity.

During post-processing, the field components *E_RE,i_* (with *i* = *x*, *y*, *z*) were retrieved using the setup’s antenna factor and cable losses, for both SSB and PDSCH, and the total electric-field strength per resource element was calculated as a vector sum of the three components. Then, the worst-case (downlink) exposure scenario for a certain user arises when all resources allocated to (PDSCH or downlink) data transmission are being used to transmit data towards the user’s UE. In that case, the resulting electric-field level *E_max_* over the whole 5G NR channel can be calculated as follows:

(1) $$E_{max}=\sqrt{f_{TDC}}\;\sqrt{12N_{RB}}\;E_{RE,PDSCH}\;[V/m],$$

with *f_TDC_* being an additional factor due to the technology duty cycle (TDC) (i.e., in the case of Time Division Duplex (TDD) the percentage of slots allocated to downlink transmission) (Table 2) and *N_RB_* the number of resource blocks (each resource block contains 12 resource elements) in the channel bandwidth [2] (Table 2).

**Step 4.** With the measurement device in frequency mode, successive traces of the power received in a 100 MHz bandwidth (i.e., the maximum bandwidth for NR signals at frequencies below 6 GHz) around the NR channel’s center frequency were captured. For the FSV setup, this measurement was repeated for each electric-field component and post-processing was necessary to average these traces to obtain the total average power over the channel bandwidth. Using the setup’s antenna factor and cable losses, the time-averaged electric-field strength, *E_avg_*, was then obtained. With the SRM setup, traces for each component were measured successively, and the resulting *E_avg_* was provided at the end by the device.

This measurement was performed twice in order to assess two extreme cases. Without an active UE, only the broadcast, control, and other background signals are measured, resulting in *E_avg,min_*. When a UE is active and maximizes the downlink traffic from the base station (see Step 3), this measurement results in a maximized time-averaged exposure level, *E_avg,max_.*

**Step 5.** Finally, the worst-case (*E_max_*) and maximum time-averaged (*E_avg,max_*) exposure levels were compared to the ICNIRP [17] exposure-limiting guidelines. The comparison to the guidelines is quantified by the exposure ratio *R*, which was calculated as follows:

(2) R=(EEref)2 [-],

with *E_ref_* the reference level (or limit for maximum permissible exposure, MPE) for the electric-field strength (i.e., 61.4 V/m for the general public and 137.3 V/m for occupational exposures) at the considered frequency, derived from *S_ref_* = 1 mW/cm^2^ for frequencies above 2 GHz [5]. The observed field levels were below the MPE if *R* ≤ 1.

### 2.3. 5G NR Base Station Sites

The measurements were performed at two NR test sites in Belgium where it was possible to measure very closely to the antennas. The parameters are listed in Table 2.

The first was a site (Site #1, Table 2) with three base station radios at a height of about 5.5 m. Each radio consisted of a 64T64R array antenna made up of 96 antenna elements in a 12-by-8 array. This base station was thus capable of massive multiple-input–multiple-output (MIMO) and beamforming and it is further denoted as an Advanced Antenna System (AAS). The second site (Site #2, Table 2) had a smaller, lower-power base station with one 4T2R antenna and thus no beamforming.

At both sites, UEs were provided by the operator: an Oppo Reno4 Pro smartphone at the first site and a Nokia 8.3 5G smartphone at the second. In both cases, the UE was connected to a 5G standalone network. To maximize the downlink traffic load (see previous section), 100 GB HTTP file downloads were set up during the measurements (from http://speedtest.tele2.net (accessed on 23 November 2021)).

It should be noted that, based on the transmit powers and EIRPs of the base stations under study, neither of them could be classified as ‘small cells’ (compare Table 1 and Table 2). However, since the exposures are directly dependent on the base station powers, the measurement results were rescaled to the respective small-cell powers, that is, to a transmit power of 30 dBm for site #1 and to an EIRP of 50 dBm for site #2 (Table 1). Moreover, a distance of 1 m to the base station was taken as boundary between workers and members of the general public (see *D_m_* for E100 in Table 1).

### 2.4. Typical Exposures

The measurement procedure of [10] summarized the above results in the *theoretical maximum* (i.e., the extrapolated worst-case) and the *measured maximum* (i.e., the maximum measured when maximizing downlink traffic) exposure levels at each measurement position. These maximum exposure levels are often reported to demonstrate compliance. However, they are not representative of typical use scenarios for most of the population. Therefore, in this study, a distinction was made between ‘worst-case users’, ‘typical users’, and ‘non-users’.

#### 2.4.1. The Worst-Case User

For a user’s worst-case exposure, two measurements were available: (1) the maximum exposure level *E_avg_*,*_max_*, measured with an active UE that maximized the base station’s downlink traffic capacity (i.e., by downloading a 100 GB file); and (2) the theoretical maximum exposure level *E_max_*, which was extrapolated based on the maximum *E_RE,PDSCH_*.

#### 2.4.2. The Typical User

Since no other use cases besides ‘downloading a 100 GB file’ were evaluated, the use case ‘making a video call,’ assessed in a previous measurement campaign [10], was selected in this study as a proxy for a ‘typical user’. In [10], in which the exposure levels were evaluated in the vicinity of a 5G NR base station with an AAS (and channel parameters CF 3.52 GHz, BW 40 MHz, and SCS 30 kHz), it was found that during a video call, the downlink (PDSCH) resource allocation was 8.4 percentage points higher than without an active UE. This indicated that, with an average baseline (i.e., without inducing any traffic) downlink resource allocation of *x*% of the total number of available resources, performing a video call increased the base station allocation of the PDSCH resources to *x* + 8.4%.

Therefore, it was assumed that the user’s exposure level during a video call can be calculated from *E_avg,min_* (baseline) and *E_max_* (100% allocation of PDSCH resources) as

(3) $$E_{avg,video\;call}=\sqrt{E_{avg,min}^{2}+0.084\;E_{max}^{2}\;f_{BW}}\;[V/m],$$

with

(4) fBW=40BW [-],

an additional factor to account for the fact that a channel with a larger bandwidth (BW, in MHz) contains more available resources.

#### 2.4.3. The Non-User

Because of 5G NR’s lean broadcast signaling, the exposure from an NR base station is almost completely dependent on the usage. Furthermore, MaMIMO techniques are used to focus the emitted energy towards the user, so non-users are effectively less exposed.

Therefore, for the exposure of a non-user, a distinction is made between the two extreme cases: (a) a non-user without any users connected to the base station under study, and (b) a non-user surrounded by a multitude of users (defined here as ‘being in a mature network’). Whereas the former’s exposure is simply *E_avg,min_*, the latter’s depends on the distribution of the users, their usage, and the MaMIMO/beamforming capabilities of the base station antenna. Both the usage and the distribution of the users are stochastic variables in space and time, and in [19], a simulation study was performed to evaluate the 95^th^ percentiles of the resulting *spatiotemporal duty cycle* (DC) (i.e., a measure of the spread of the base station’s emitted power in space and time) of base station radios with varying antenna array sizes and thus varying MaMIMO capabilities. In general, the higher the number of antenna elements in the array, the lower the DC, as MaMIMO capabilities increase, e.g., to form more narrow beams directed towards users and away from non-users. For this study, the assumptions in Table 3 were adopted, for which *DC_site_*_#1,95_ = 0.20 and *DC_site_*_#2,95_ = 0.96 were found in [19]. Assuming fewer active users and/or shorter typical usage, lower DCs were also found in [19]. However, a more detailed analysis was out of the scope of this study.

As the site-specific spatiotemporal DC is a stochastic measure for the percentage of time a non-user is actually exposed by a base station servicing numerous active users. The exposure level *E_avg,non-user with other users_* is then calculated as

(5) $$E_{avg,non-user\;with\;other\;users}=\sqrt{DC_{site,95}\;}E_{avg,max}\;[V/m],$$

with *E_avg,max_* representing the exposure of a single user demanding as many of the base station’s downlink resources as possible and *DC_site_*_,95_ the 95th percentile of the spatiotemporal DC obtained in [19] for the specific site parameters (number of antenna elements and MaMIMO scheme, Table 3). Adding the DC in Equation (5) effectively accounts for the spreading of the base station’s (maximized) radiated power over the area it services.

## 3. Results

**Figure 3:**
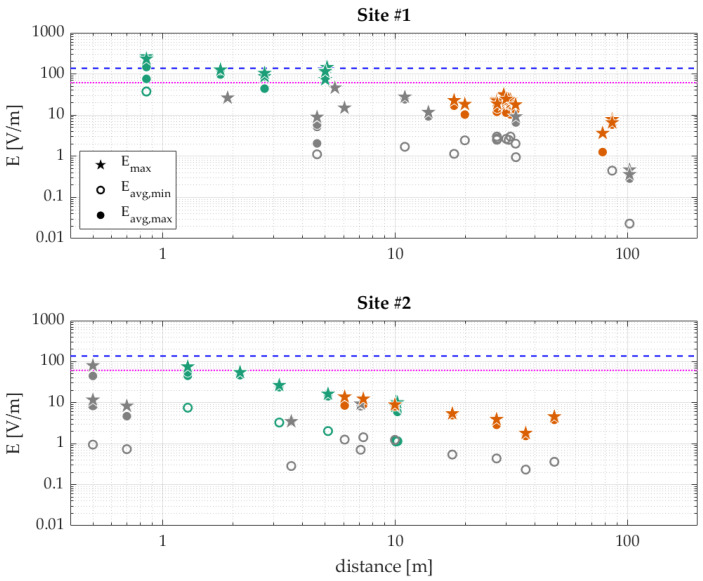
Three types of electric-field levels (*E_avg,min_* as rings, *E_avg,max_* as circles, and *E_max_* as pentagons; all in V/m) measured at two sites with a 5G new radio (NR) base station as a function of the distance to the antenna with two types of measurement equipment, namely an FSV setup (green markers) and an SRM setup (orange markers) (markers in grey denote measurements outside the scanning range of the base station radio and were added for completeness rather than direct comparison). Maximum permissible exposure (MPE) limits issued by ICNIRP [17] are indicated by the blue dashed line (occupational) and the magenta dotted line (general public).

The three types of exposure levels obtained directly from the measurements (the extrapolated worst-case *E_max_*, and the time-averaged *E_avg,min_* and *E_avg,max_*) are shown in Figure 3 for both sites as a function of distance to the base station.

In total, with the SRM setup, measurements were performed at 30 positions at horizontal distances from the base station ranging from 0.5 m to 100 m, all at 1.5 m above the ground or floor (three positions were performed on the flat roof of the second site), and with the FSV at 12 positions at horizontal distances between 0.9 m and 10 m, with the probe on a telescopic pole placed at a height between 2.2 m to 5.5 m. Most of the measurements were within the scanning range of the base station radio. Those that were not (indicated by grey markers in Figure 3), were either too close to the base station (horizontally) so they were below the main lobe of the radiation pattern, or too far to the sides of the radiation pattern.

First of all, the results of the FSV and SRM measurement setups were visibly in line with each other. This was observed most clearly at site #2, where there was an overlap in the distances at which measurements were taken. Hence, the measurement results of the two setups were treated as equivalent.

Furthermore, it is observed that the exposure levels at positions outside the scanning range (e.g., below the antenna) were 25–100 times lower than those within the scanning range at the same distance (Figure 3).

**Figure 4:**
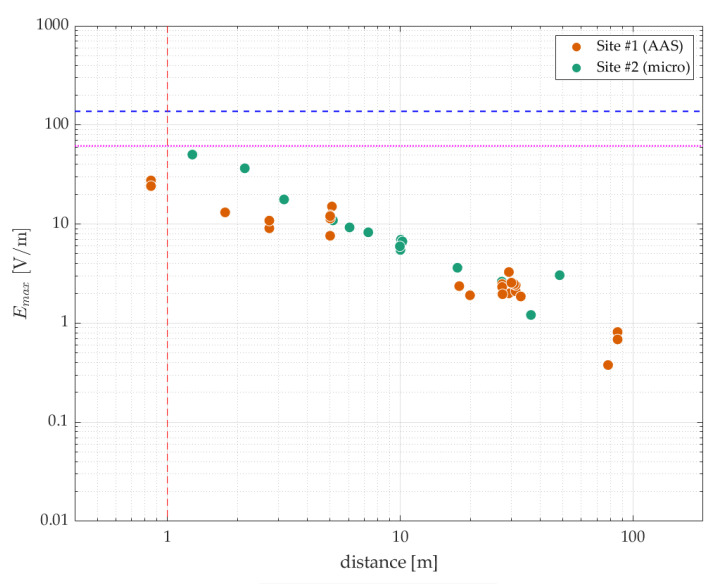
Theoretical maximum exposure levels *E_max_* (in V/m) obtained at two sites with a 5G new radio (NR) base station—scaled to small-cell powers [2,3]—as a function of the distance to the antenna. Only positions within the scanning range of the base station radio are featured here. Maximum permissible exposure (MPE) limits issued by ICNIRP [17] are indicated by the blue dashed line (occupational) and the magenta dotted line (general public). The red dashed line indicates the boundary between occupational (<1 m) and general public exposure (>1 m) (see *D_m_* for E100 in Table 1).

In Figure 4, the theoretical maximum exposure levels *E_max_* obtained at the two sites were scaled to the respective small-cell powers, i.e., to an EIRP of 50 dBm for site #2 (E100 microcell) and to a transmit power of 30 dBm for site #1 (AAS small cell). Between the two sites, the exposure levels are very similar, especially at distances above 5 m. However, closer to the base station, the scaled levels at site #2 were higher, despite the lower EIRP. The flatter log(distance)–log(electric-field strength) curve of site #1 at short distances may be due to the more extensive radiating near-field region of its larger AAS (up to ~20 m).

Scaled to the respective small-cell powers, the maximum time-averaged electric-field levels (*E_avg,max_*) measured during maximum traffic load were 27.5 V/m (at a distance of 0.8 m from the center of the antenna) and 50.2 V/m (at 1.3 m) for the AAS and microcell base stations, respectively (Figure 4). Both levels are below the MPE limits set by ICNIRP, which are 61.4 V/m for general public exposure and 137.3 V/m for occupational exposure for frequencies in the n78 band [17]. Considering a cut-off of 1 m separation distance between the base station and humans, the highest exposure ratios were 0.15 for occupational exposure and 0.68 for exposure of the general public, both of which were found for site #2 (with an EIRP of 50 dBm).

Furthermore, Table 4 lists the minimum (*E_avg,min_*) and worst-case exposure levels (*E_max_* and *E_avg,max_*) for workers and members of the general public, as well as the interpolated exposure levels by considering typical usage (video call, Equation (3)) and the exposure levels for non-using bystanders, using the spatiotemporal DCs of [19] for the case with numerous randomly distributed users in a mature network (Equation (5)).

The values in Table 4 offer a range of the highest exposure levels that can be expected at small-cell sites, both for workers at a distance of minimum 0.5 m from an operational base station (data at <0.5 m are not available) and for members of the general public at a distance of ~1.3 m (data at 1–1.3 m are not available). At positions further away than those at which these maxima were observed, the exposure levels in all categories of Table 4 were lower (see also Figure 3 and Figure 4). In any case, all values were below the MPE limits of [17].

In general, the theoretical worst-case exposures are higher than the maxima observed in situ by maximizing the downlink traffic load [13,18]. Both worst-case exposures are much higher than the exposures of a typical (solitarily connected) user, which are in their turn much higher than the exposures without any users. The exposure of a non-user to other users depends on the distribution of the other users, their usage, and the AAS capabilities of the base station radio.

Even though NR signaling is much leaner than its predecessors’, *E_avg,min_* was not negligible close to the base station (fourth row in Table 4). Furthermore, the exposure levels quickly increased when using a device connected to the base station, even for a video-calling user (in that case by a factor of 4 to 9), although it remains a fraction of the worst-case exposure levels (in that case ~6%). In general, the theoretical worst-case exposures were higher than the maxima measured in situ when maximizing the downlink traffic load. Finally, in a mature 5G NR network with an average of 10 simultaneous users, a non-user’s exposure from an AAS base station with 96 antenna elements could be five times lower than that of a worst-case user, while their exposure from a non-AAS base station would be about the same as the users’ (compare the last row with the first two in Table 4).

## 4. Discussion and Conclusions

This paper describes radiofrequency (RF) electromagnetic field (EMF) measurements in the vicinity of two 5G new radio (NR) base stations, both transmitting in the 5G NR n78 band (3.4–3.8 GHz), scaling the results to small-cell base station powers and the extrapolation of the obtained field levels to different exposure cases.

The measurements themselves consisted of evaluating the worst-case field levels as well as measuring the time-averaged field level without any induced downlink traffic load and when maximizing the downlink traffic load (from the base station to the user device), at positions at various distances from the base stations, following IEC 62232 [2]. Then, in order to evaluate the exposures potentially induced by 5G NR small-cell base stations, the results were scaled to base station radio transmit powers maximally used by small cells [2,3]. Furthermore, these measurements were used to calculate the exposure levels for a typical user (making a video call) and a non-user in a mature network.

Scaled to small-cell powers, the measured exposure levels in this study were below the MPE limits for both occupational (at distances between 0.5 m and 1 m from the base station) and general public exposure (>1 m) issued by the International Commission on Non-Ionizing Radiation Protection (ICNIRP) [17]: the maximum exposure ratios were 0.15 (occupational) and 0.68 (general public). These theoretical worst-case exposures were higher than the actual maxima measured in situ by maximizing the downlink traffic load, and both types of worst-case exposures were much higher (3–12 times) than the exposures of a typical user, which in turn were much higher (4–9 times) than the exposures without any users. Finally, the exposure of a non-user within a mature 5G NR network depends on the distribution of users, their usage, and the AAS capabilities of the base station radio.

Although adverse health effects at non-thermal exposure levels cannot be ruled out [20], the ICNIRP reference levels are still relevant to calculate exposure ratios against. The measurement values obtained in this study and reported in this paper can be directly compared to other reference or limit levels (e.g., from legislation or scientific literature), depending on the scope of the study.

For a 5G NR AAS base station, the actual exposure of a given user will generally be less than the theoretical maximum exposure *E_max_* for several reasons. First, other users (the number may vary) may be in beams other than the one the given user is using. Hence, the RF energy directed toward these users will not add (much) to the exposure of the given user. Second, the usage by the given user will generally be less than the maximum assumed for which *E_max_* was defined. Third, there may be dynamic power control to reduce base station power to the minimum needed for communication. This was not taken into account in this study. Finally, the base station beam may be narrower or wider than that used to measure *E_max_*. Hence, the problem of determining actual RF exposure becomes a statistical one that depends on several different variables. However, the exposure will almost always be less than *E_max_*.

Similarly, for the non-user, the difference lies in whether they are in a beam or not. Generally, the RF exposure from the base station for the non-user will be smaller than that for a user (here by at least a factor of 5 for an AAS), unless there are many users around and the MaMIMO capabilities of the base station are limited (Table 4) [19].

The fact that the measurement results of the FSV and SRM setups are similar (Figure 3) is an important result, as it indicates that the less cumbersome SRM measurement setup performed adequately, if used in a correct way as described here, and can thus be used in future 5G NR measurement campaigns for *E_RE_* (and *E_max_* extrapolation, as described in [10]) and *E_avg_* measurements.

Furthermore, the results of this work can be used by, e.g., electric utilities who are considering placing 5G small cells on poles or other utility infrastructure in neighborhoods. In communicating with the public about this possibility, it is helpful if utilities are shown to be proactive in research on RF exposure. Furthermore, inquiries about possible RF exposure can be answered with more confidence (even though the particular situations might be different) if measured exposure levels are available. The maximum exposure levels reported here are generally applicable at other sites. As they are directly dependent on the power of the base station, they can easily be scaled to the base station powers at these sites. For example, for the E10 base station class [2,3], the 10 dB (i.e., tenfold) decrease in maximum power compared to E100 (Table 1) would result in maximum exposure ratios of 0.02 (occupational) and 0.07 (general public). Furthermore, the results of the E100 base station at site #2 should be valid at any site with a passive antenna with the same antenna gain.

Finally, due to their physical properties (larger available bandwidth, smaller antenna arrays, and the fact that small cells would be in the line of sight of many users anyway), the use of millimeter waves (‘mmWaves’), with frequencies above 24 GHz, may be preferred for 5G NR small cells, compared to the sub-6 GHz frequencies. However, due to the availability of sites for the study duration, for this paper only 5G NR signals at sub-6 GHz frequencies were considered. Future work will consist of analyzing the exposure levels of small cells operating at much higher frequencies (e.g., 26 GHz), using similar methods as described in this paper (with settings adjusted according to the different frequency, bandwidth, and signal structure) and discussed in [14,15,16].

## Figures and Tables

**Table 1:** The IEC’s simplified safe installation criteria for base station classes [2]. Classes E100, E10, and E2 fall under the general definition of a small cell.

Class	EIRP [dBm]	Product Installation Criteria
E2	≤33	[…] Compliance with the exposure limits is generally obtained at zero distance or within a few centimeters.
E10	≤40	[…] the lowest radiating part of the antenna(s) is at a minimum height of 2.2 m above the general public walkway.
E100	≤50	[…] (a) the lowest radiating part of the antenna(s) is at a minimum height of 2.5 m above the general public walkway; (b) the minimum distance to areas accessible to the general public in the main lobe direction is D_m_; (c) there are no pre-existing RF sources with EIRP above 10 W installed within a distance of 5 D_m_ meters in the main lobe direction (as determined by considering the half power beam width) and within Dm meters in other directions. If D_m_ is not available, a value of 2 m can be used or 1 m if all product transmit frequencies are equal to or above 1500 MHz.
E+	>50	[…]

**Table 2:** Parameters of the 5G new radio (NR) base station radios at the two considered sites in Belgium.

	Site #1	Site #2
Frequency band	n78 (FR1)	n78 (FR1)
Channel center frequency	3.775 GHz	3.430 GHz
Channel bandwidth	50 MHz	40 MHz
Subcarrier spacing	30 kHz	30 kHz
Maximum number of resource blocks (*N_RB_*)	133	106
Number of antenna elements (per polarization)	96	4
MIMO	64T64R	4T2R
Advanced Antenna System (AAS)?	Yes	No
Transmit power	49.7 dBm (92.5 W)	43 dBm (20 W)
Maximum gain (array gain + antenna element gain)	25 dBi	10.5 dBi
EIRP	74.7 dBm (29.5 kW)	53.5 dBm (112 W)
Height	5.5 m	4.5 m
Size of radio unit	795 mm × 470 mm × 190 mm	295 mm × 270 mm × 20 mm
Technology duty cycle factor (*f_TDC_*)	0.743	0.743

FR1 = Frequency Range 1 (410 MHz to 7125 MHz), MIMO = multiple-input–multiple-output, EIRP = equivalent isotropic radiated power.

**Table 3:** Assumptions made in the calculation of the spatiotemporal duty cycles at the two 5G new radio (NR) sites considered in this study [19].

Variable	Site #1	Site #2
Number of active users	10	10
Typical usage (connection time)	10 s	10 s
Number of antenna elements	100	4
MaMIMO/beamforming scheme	Codebook ‘grid of beams’	n/a
*DC_site_*_,__95_	0.20	0.96

n/a = not applicable.

**Table 4:** Summary of maximum worst-case and maximum typical exposure levels (*E*, in V/m) and corresponding exposure ratios *R* (Equation (2)) for an Advanced Antenna System (AAS) small-cell base station (at site #1) and a E100 small-cell base station (site #2), found for workers (at a distance between 0.5 m and 1 m from an operational base station) and members of the general public (at a distance of >1.3 m).

User Type	Electric-Field Level *E* (V/m) and Exposure Ratio *R (-)*			
	Workers		General Public	
	*AAS*	*E100 ^(1)^*	*AAS*	*E100*
**Worst-case user** *(maximum exposure, with base station at maximized downlink traffic capacity)*	21.6 (0.03)	30.0 (0.05)	12.8 (0.04)	36.6 (0.36)
**Worst-case user** *(worst-case exposure, extrapolated E_max_)*	27.5 (0.04)	53.6 (0.15)	13.1 (0.05)	50.8 (0.68)
**Typical user** *(performing a video call)*	7.8 (0.003)	16.5 (0.02)	4.2 (0.005)	15.5 (0.06)
**Non-user without other users** *(no induced downlink traffic, E_avg,min_)*	3.9 (0.0008)	5.7 (0.002)	2.4 (0.002)	5.0 (0.007)
**Non-user in mature network** *(based on maximum exposure, with base station at maximized downlink traffic capacity, and a 95th percentile spatiotemporal duty cycle to account for spatiotemporal dispersion of power)*	9.7 (0.005)	29.4 (0.05)	5.7 (0.009)	35.9 (0.34)

Additional information is added (in italics) for each of the considered user types (names in bold). The ICNIRP reference levels used to calculate the exposure ratios (Equation (2)) were 61.4 V/m for the general public and 137.3 V/m for workers [17]. ^(1)^ At site #2, no measurements were performed at distances closer than 1 m within the scanning range of the base station, only to the sides of the antenna.

## Data Availability

The data presented in this study are available on request from the corresponding author.
